# Absence of Auditory M100 Source Asymmetry in Schizophrenia and Bipolar Disorder: A MEG Study

**DOI:** 10.1371/journal.pone.0082682

**Published:** 2013-12-10

**Authors:** Ying Wang, Yigang Feng, Yanbin Jia, Yanping Xie, Wensheng Wang, Yufang Guan, Shuming Zhong, Dan Zhu, Li Huang

**Affiliations:** 1 Medical Imaging Center, First Affiliated Hospital of Jinan University, Guangzhou, China; 2 Clinical Experimental Center, First Affiliated Hospital of Jinan University, Guangzhou, China; 3 Medical Imaging Center, Guangdong 999 Brain Hospital, Guangzhou, China; 4 Department of Psychiatry, First Affiliated Hospital of Jinan University, Guangzhou, China; VU University Medical Center, Netherlands

## Abstract

**Background:**

Whether schizophrenia and bipolar disorder are the clinical outcomes of discrete or shared causative processes is much debated in psychiatry. Several studies have demonstrated anomalous structural and functional superior temporal gyrus (STG) symmetries in schizophrenia. We examined bipolar patients to determine if they also have altered STG asymmetry.

**Methods:**

Whole-head magnetoencephalography (MEG) recordings of auditory evoked fields were obtained for 20 subjects with schizophrenia, 20 with bipolar disorder, and 20 control subjects. Neural generators of the M100 auditory response were modeled using a single equivalent current dipole for each hemisphere. The source location of the M100 response was used as a measure of functional STG asymmetry.

**Results:**

Control subjects showed the typical M100 asymmetrical pattern with more anterior sources in the right STG. In contrast, both schizophrenia and bipolar disorder patients displayed a symmetrical M100 source pattern. There was no significant difference in the M100 latency and strength in bilateral hemispheres within three groups.

**Conclusions:**

Our results indicate that disturbed asymmetry of temporal lobe function may reflect a common deviance present in schizophrenia and bipolar disorder, suggesting the two disorders might share etiological and pathophysiological factors.

## Introduction

Historically schizophrenia and bipolar disorder have been considered two separate diagnostic entities since Kraepelin’s nosologic distinction. Schizophrenia is presumably a neurodevelopmental disorder characterized by hallucinations, delusions, and cognitive deficits. Bipolar disorder is characterized by recurrent episodes of mania and depression, but is also often accompanied by cognitive deficits and psychotic symptoms. Recent epidemiological and genetic studies have suggested that schizophrenia and bipolar disorder have certain overlapping etiological factors [1], or share several chromosomal loci and genes[2,3]. Furthermore, there is growing evidence of similarity in the pattern of cognitive and neurobiological deficits in schizophrenia and bipolar disorder [4,5]. These findings offer a substantial challenge to the traditional Kraepelinian model of the two disorders as having fully discrete underlying disease processes. However, it has been recommended that the Kraepelinian dichotomy not be hastily abandoned, and the final decision should be made very cautiously [6,7,8]. Therefore, further analysis and description of the underlying neurobiological processes of both illnesses would be valuable.

These asymmetries, also referred to as laterality, are known to be present in the normal human brain. In fact, anomalous lateralization has been promoted as a centrepiece for a theory of schizophrenia involving language and human speciation [9]. Disturbances in cerebral functional and structural asymmetries are a well-documented finding in schizophrenia patients and specific brain areas, like the temporal lobe, are particularly affected. Structural neuroimaging studies have shown reversals or losses of normal anatomic asymmetry of the planum temporale [10], superior temporal gyrus (STG) [11] and sylvian fissure [12] in schizophrenia, particularly through changed in the left temporal lobe [13]. It has been postulated that the genetic mechanism underlying normal left hemispheric dominance is altered in schizophrenia [13]. Functional neuroimaging studies have also reported reduction in functional asymmetry, for example, decreased language lateralization on the dichotic listening paradigm [14]. In addition, disturbed neurochemical asymmetries have also been reported in the temporal lobe of patients with schizophrenia [15]. However, there are relatively fewer reports on altered anatomical and functional asymmetries in bipolar disorder compared with the extensive efforts applied in schizophrenia.

Magnetoencephalography (MEG) is a noninvasive functional neuroimaging technique for investigating neural activity in the living human brain. The strengths of MEG are its temporal and spatial accuracy. Unlike indirect measures such as functional magnetic resonance imaging (fMRI), positron emission tomography (PET) or single-photon emission computed tomography (SPECT), which all record aspects of brain blood ﬂow or metabolism, MEG records neuronal activity directly and thus records real-time activity with millisecond resolution [16,17]. MEG is suitable for overcoming the methodological limitations of fMRI, such as low temporal resolution and the influence of the noisy environment from surrounding devices. On the other hand, electroencephalography (EEG) and MEG are closely related, in principle both reﬂecting the same neuronal currents. However, EEG quantification as compared to MEG depends on a recording ‘reference’ point. Different references will lead to different results [18]. Moreover, MEG allows monitoring of cortical activation sequences without severe distortion by the resistive properties of the skull and scalp [16,19]. Thus, MEG— with its good temporal resolution and advantages over EEG— is gaining a well-established role in neuropsychiatric imaging research, complementing the spatially more focused hemodynamic fMRI technique [19].

Neuromagnetic responses in the auditory cortex to an auditory stimulus, termed auditory evoked fields (AEFs) include several components, such as the M50 (magnetic analog of the evoked potential P50), M100 (magnetic analog of the N100), M200. The M100, a wave peaking around 100 ms after the stimulus onset, has been considered the most prominent response in the auditory system in adults. It represents the tangential part of the supratemporal auditory electric N1 component. In healthy adults, the neuroanatomic source of the auditory M100 exhibit interhemispheric asymmetry, being further anterior in the right relative to the left hemisphere [20]. However, patients with schizophrenia have been found to demonstrate a reduction or even reversal of interhemispheric asymmetry of several AEF source locations, including the M50 [21] and M100 [22-25]. Moreover, these disturbance asymmetries may be more pronounced in male than in female patients [22]. However, very few investigations have been focusing on AEFs asymmetries of bipolar disorder. Only one study reported abnormal laterality of auditory cortex in euthymic bipolar subjects [26].

In the current study, a whole-head MEG device was employed to investigate functional asymmetries in schizophrenia and bipolar disorder patients. The source location of auditory M100 response was used as a measure of functional temporal lobe asymmetry. Our hypothesis is that patients with bipolar disorder will show anomalous M100 hemispheric asymmetry similar to that showed in schizophrenia. We believe such data will contribute to our improved understanding of the relationship between bipolar disorder and schizophrenia on the basis of objective physiological measurements.

## Materials and Methods

### 2.1: Subjects

Twenty patients with schizophrenia and 20 patients with bipolar disorder were recruited from the in-patient unit of the psychiatry department, First Affiliated Hospital of Jinan University, Guangzhou, China. The patients were aged from 18 to 60 years. All patients were diagnosed with either schizophrenia or bipolar disorder by a trained psychiatrist using the Structured Clinical Interview for the DSM-IV-Patient Edition (SCID-P) and DSM-IV criteria. Exclusion criteria included the presence of (1) other Axis I psychiatric disorders and symptoms, (2) a history of electroconvulsive therapy (ECT), (3) a history of organic brain disorder, neurological disorders, or cardiovascular diseases, (4) alcohol/substance abuse within 6 months before study entry, and (5) pregnancy or any physical illness demonstrated by personal history, or clinical or laboratory examinations. All subjects with schizophrenia or bipolar disorder were medicated. None of the bipolar patients were currently suffering from psychotic symptoms. Clinical symptoms were assessed by use of the Positive and Negative Syndrome Scale (PANSS) for patients with schizophrenia, the Young Mania Rating Scale (YMRS) and the Hamilton Depression Rating Scale (HDRS) (17-item version) for patients with bipolar disorder. 

Twenty healthy control subjects were also recruited via local advertisements. They were carefully screened through a diagnostic interview, the Structured Clinical Interview for DSM-IV Nonpatient Edition (SCID-NP), to rule out the presence of current or past psychiatric illness. Further exclusion criteria for healthy controls were any history of psychiatric illness in first-degree relatives, current or past significant medical or neurological illness, and hearing impairment.

All participants were nonsmokers, good hearing (at least 60 dB in each ear), and right-handed. No subjects required sedation for scanning. The study was approved by the Ethics Committee of First Affiliated Hospital of Jinan University, China. All subjects signed a written informed consent form after a full written and verbal explanation of the study. Two senior clinical psychiatrists confirmed that all subjects had the ability to consent to participate in the examination.

### 2.2: Neuroimaging data collection

#### 2.2.1: Stimuli

Auditory stimuli consisting of 2 kHz tones, 30 ms duration (5 ms rise and fall times), 1s inter-stimulus intervals (ISI) and 80 dB sound pressure level (SPL), were generated with BrainX software [27] and delivered to the subject binaurally through the plastic tubes with plastic insert earpieces at the tip. A total of 100 stimuli was presented to ears. Participants were instructed to listen to the tones carefully without any task and to minimize the eye blinking. A video camera installed inside the chamber allowed monitoring the subject's behavior and compliance at any time throughout the experiment.

#### 2.2.2: MEG and MRI data collection

Studies were performed using a whole-head Magnes 2500 WH (148 channel) magnetometer (4D Neuroimaging, San Diego, CA), a helmet shaped dewar covering the entire adult head, except the face. The subjects lay on the positioning bed inside the magnetically shielded room and auditory stimuli were presented to each ear. Three small electrode coils, used to transmit subject location information to the neuromagnetometer probe, were taped to the forehead with two-sided tape. Two electrode coils were taped in front of the right and left preauricular point. These coils provide for specification of the position and orientation of the MEG sensors relative to the head. A 3D digitization system was used to determine the subject’s head shape in a head centered coordinate system defined by the nasion and right and left preauricular points. The X-axis defined anterior–posterior directions, Y defined the right and left directions, and Z defined superior–inferior directions. Activation of these electrode coils before and after each study allowed the localization of the MEG measurement array with respect to the subject's head. The shape of the head was also digitized for help with later coregistration to a standard MRI scan. The MEG was recorded with a 678.17 Hz sampling rate, using a bandpass filter of 0.1-200 Hz. Recordings included a 100msec prestimulus baseline and 500msec following stimulus delivery.

After the MEG session, structural magnetic resonance imaging (MRI) provided T1-weighted, three-dimensional (3D) anatomic images using the Gyroscan Intera 1.5T (Philips Medical Systems, The Netherlands). The pulse sequence was a T1-weighted 3 D fast field echo (FFE) with the following parameters: TR=25 ms, TE=4.6 ms, ﬁeld of view =240 mm, flip angle=30°, matrix 256*256, slice thickness=1.2 mm, no gap, 140 slices obtained in 3 min 16 se. Three points were marked on the nasion and bilateral preauricular points to be visualized on MRI images with small oil-containing capsules (3 mm diameter). T1- weighted images (axial, coronal and sagittal slices) were used for overlays, with the equivalent current dipole sources detected by MEG.

#### 2.2.3: MEG data processing and source localization

The data were collected and analyzed using a software package (MSI software, WHS version 1.2.4, Biomagnetometer system) on a workstation (SUN, SPARC Station™). In the off line analysis, the MEG was triggered by stimulus onset and it was averaged for each condition. A 1–40 Hz bandpass filter was applied to each subject’s cross-trial-averaged MEG data. The M100 peak latency was defined as the latency with the largest amplitude within the time window of 80--150 ms post stimulus. A single equivalent current dipole model was adopted for MEG source analysis, which assumes that the neuronal sources were focal. Dipolar sources were identified in the left and right hemisphere for M100 responses to the auditory stimulus. Determination of the location, peak strength, and latency of the M100 sources in the bilateral hemispheres were accomplished by fitting each dipole using 34–37 channels separately over the left and right temporal lobe. Only equivalent current dipoles with goodness-of-fit values (a measure of the correlation between calculated and measured signal) exceeding 90% were accepted for further analysis. In the study, all subjects met the criteria of goodness-of-fit > 90%. Peak strength of the source over the 10-ms period was then determined. MEG data were superimposed over T1-weighted structural MRI images for data coregistration. The coordinates of these MRI-based measures were aligned with the MEG-coordinate system by identifying the left and right preauricular points, as well as the nasion, from the MR scans. These measures were conducted by the same trained investigator, who was blind to each subject diagnosis.

### 2.3: Statistical analysis

All data analyses were performed using SPSS for Windows software, version 15.0 (SPSS Inc., Chicago, III, USA). The alpha criterion for non-significance was>0.05. All significance tests were two-tailed. One-way analyses of variance (ANOVAs) and chis-quare tests were used to assess group differences for continuous and categorical demographic variables. Group differences in M100 source locations (x, y, and z codes), latency and strength were analyzed using two-way ANOVAs with group (schizophrenia, bipolar disorder, and control) as the between subjects factor and hemisphere (left and right) as the within subjects factor. The asymmetry was determined using paired sample *t*-tests of left and right hemispheric M100 source locations (x, y, and z codes) for each group. Asymmetry indexes were also calculated using the formula (R - L)/(R + L) ×100 for direct evaluation of differential source lateralization among three groups. Data were presented as means and standard deviations. Pearson’s correlation coefficients were used to correlate clinical variables to the measured M100 parameters of location, latency, and strength.

## Results

The demographic and clinical characteristics of the three groups are summarized in Table 1. There was no significant difference of the subjects recruited in this study in age, sex, and length of education among the three groups.

**Table 1:** The demographic characteristics of the subjects of three groups.

	**Schizophrenia**	**Bipolar disorder**	**Control**
	(n=20)	(n=20)	(n=20)
**Gender (male/female)**	10/10	9/11	8/12
**Age (years)**	34.10 (12.03)	29.40 (7.33)	34.84 (11.07)
**Education (years)**	13.40 (3.13)	15.53 (2.09)	14.47 (2.44)
**Duration of illness (years)**	5.44 (3.79)	2.57 (2.01)	n/a
**PANSS score (points)**	98.33 (9.38)	n/a	n/a
**HDRS score (points)**	n/a	23.60 (4.98)	n/a
**YMRS score (points)**	n/a	13.95 (7.86)	n/a

Standard deviations are in parentheses. PANSS, Positive and Negative Syndrome Scale; YMRS, Young Mania Rating Scale; HDRS, Hamilton Depression Rating Scale.

**Figure 1:**
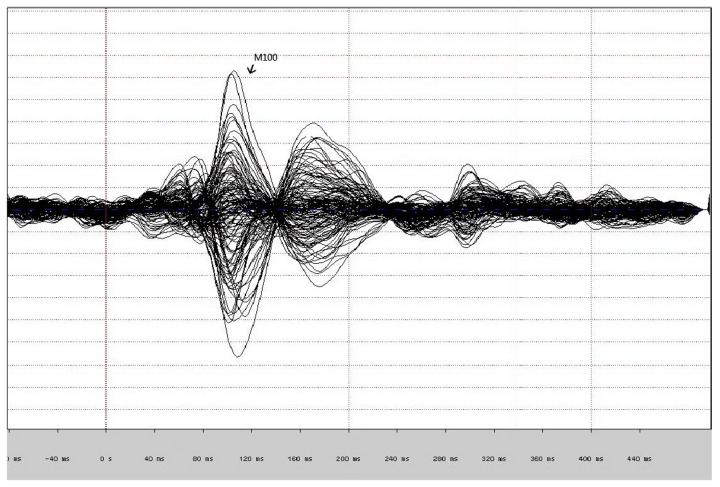
Averaged neuromagnetic responses (overlay of all 148 channels) to the auditory stimuli in the control subject.

**Figure 2:**
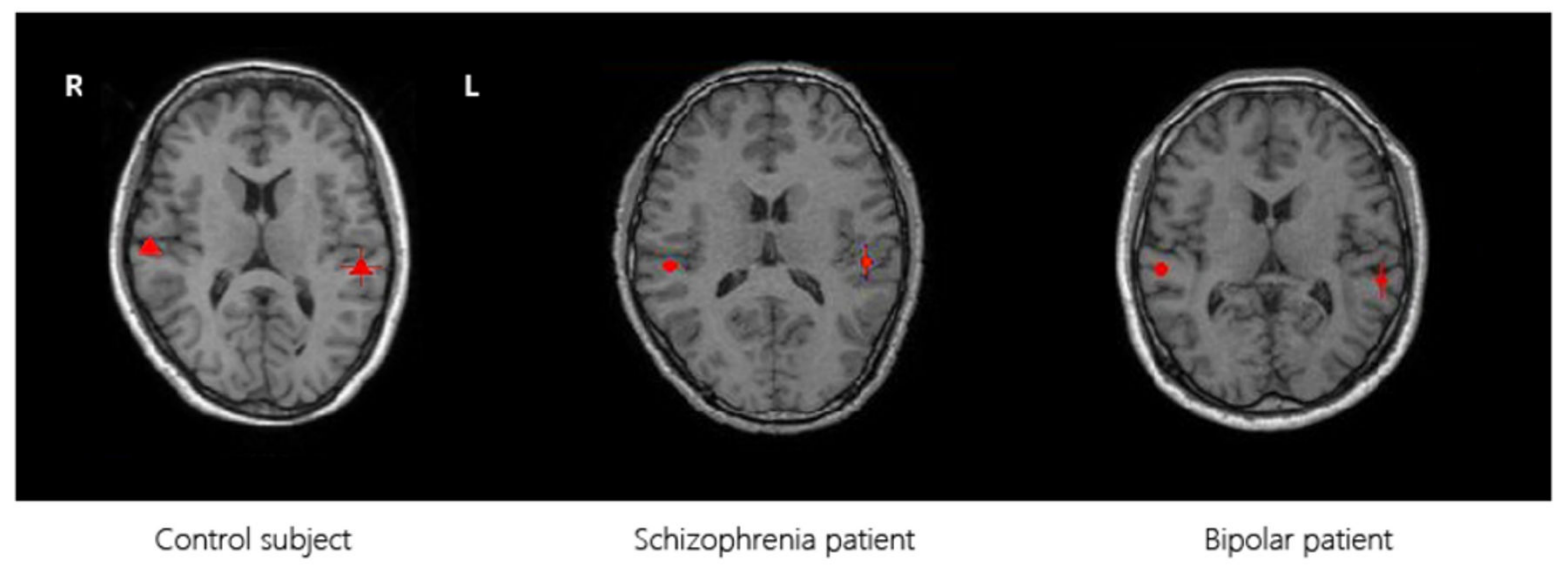
Locations of M100 dipole sources in an individual control subject, an individual schizophrenia, and an individual bipolar disorder on axial T1- weighted images.

**Figure 3:**
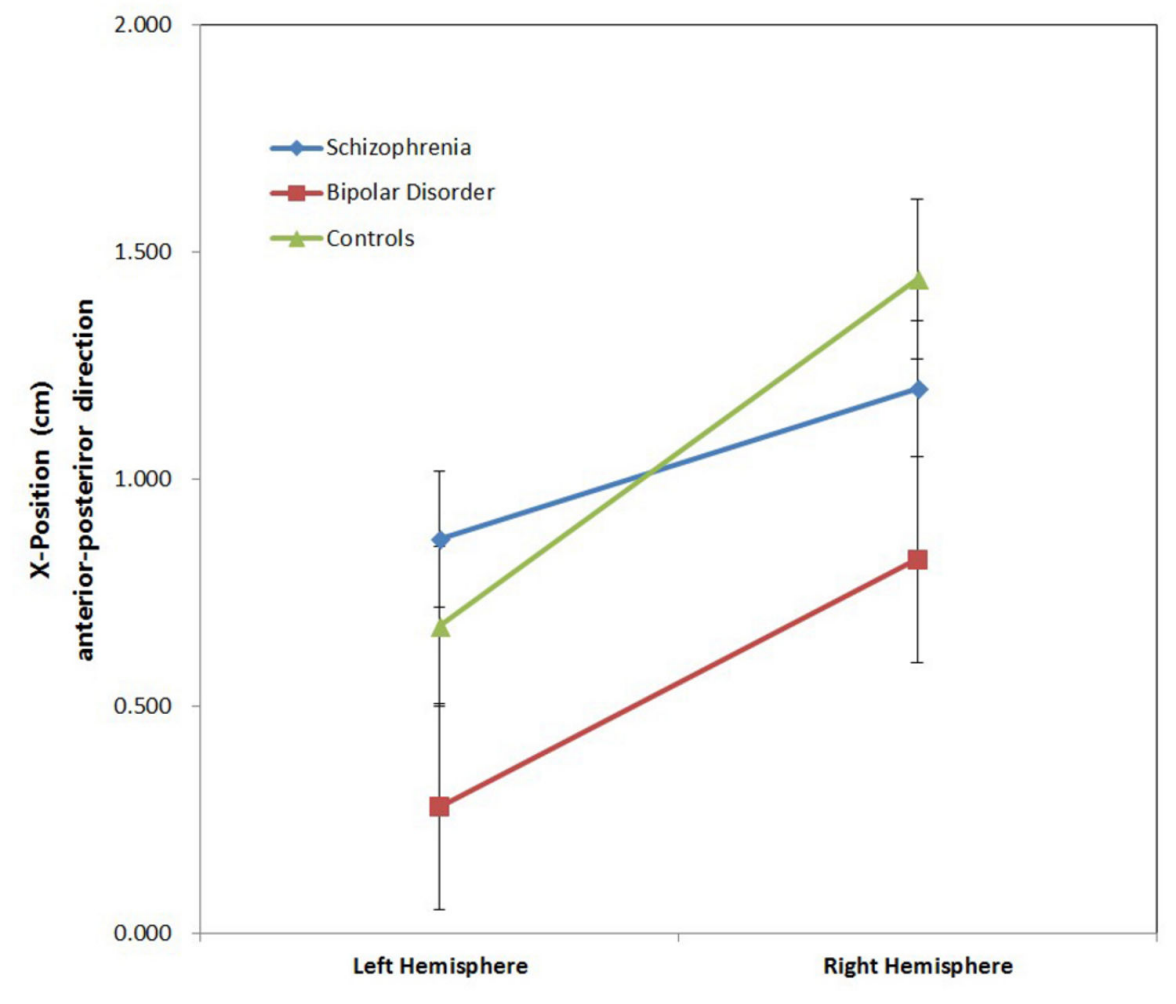
M100 anterior–posterior (X) positions: average data (with standard error bars) are shown for the three subject groups (controls, subjects with schizophrenia, subjects with bipolar disorder). As shown, right-hemispheric sources were significantly anterior to those of the left only in controls (*p* < 0.01, paired *t*-test).

Figure 1 provides an example of averaged neuromagnetic responses of M100 (overlay of all 148 channels) to the auditory stimuli for one subject. In every subject, M100 responses were detected, and M100 sources localized to the left and right posterior portion of STG or near primary auditory cortex (Figure 2). The M100 source locations for all subjects in terms of X (posterior–anterior), Y (medial–lateral), and Z (inferior–superior) values are shown in Table 2. In control subjects, X values M100 for sources in right are significantly more anterior than X values M100 for sources in left (*t* = -8.05, *p* = 0.000). However, this anterior–posterior asymmetric pattern failed to present in schizophrenia (*t* = -1.32, *p* >0.05) and bipolar disorder (*t* = -1.87 *p* > 0.05). Figure 3 illustrates the mean anteroposterior dipole locations for each group. For the M100 source localization X values, two-way ANOVAs with group (schizophrenia, bipolar disorder, and control) and hemisphere (left and right) as factors showed significant main effects of group (*F* (2, 57) = 4.692, *p* = 0.011), and hemisphere (*F* (1, 57) = 14.490, *p* =0.000), but a group × hemisphere interaction was not observed (*F* (2, 57) = 0.902, *p* =0 .409). Post hoc analysis revealed that the M100 source was generally located more anterior in the schizophrenia group (*p* = 0.013) and no difference between the bipolar group (*p* = 1.000) compared with the control group. However, post hoc tests revealed a significant difference between the schizophrenia group and bipolar group (*p* =0.040). No significant main or interaction effects were observed for the M100 source localization Y and Z values. Moreover, one-way ANOVA revealed no significant difference in M100 asymmetry indexes for the X, Y and Z coordinate among groups. 

**Table 2:** M100 source locations (X: posterior–anterior, Y: medial–lateral, Z: inferior–superior) for each group.

	**X**			**Y**			**Z**		
	**Left (mm)**	**Right (mm)**	**AI**	**Left (mm)**	**Right (mm)**	**AI**	**Left (mm)**	**Right (mm)**	**AI**
**Control**	6.75 (7.35)	14.40 (6.25) *	88.90 (254.29)	54.46 (5.54)	-54.09 (6.40)	-0.42 (9.17)	55.58 (6.56)	55.97 (6.05)	0.23 (6.30)
**Schizophrenia**	8.66 (6.85)	11.99 (5.23)	27.52 (69.16)	56.49 (7.05)	-54.89 (5.72)	-1.31 (8.86)	55.55 (5.39)	54.69 (7.70)	-0.94 (5.68)
**Bipolar disorder**	2.78 (7.87)	8.23 (8.49)	50.90 (150.82)	52.85 (7.05)	-53.94 (6.38)	0.53 (9.96)	55.54 (6.26)	57.57 (5.78)	1.99 (3.47)

Standard deviations are in parentheses. AI, Asymmetry Index. (* *p* < 0.01, paired *t*-test).

With regard to the M100 latency, no main effects of group (*F* (2, 57) = 0.554, *p* = 0.644) or hemisphere (*F* (1, 57) = 0.001, *p* = 0.982), or significant group × hemisphere interaction (*F* (2, 57) = 0.598, *p* = 0.552) were found. For the M100 strength, results showed no main effects of group (*F* (2, 57) = 9.418, *p* = 0 .096) or hemisphere (*F* (1, 57) = 0.002, *p* = 0.971), or interaction (*F* (2, 57) = 0.292, *p* = 0.748) (Table 3). Furthermore, there was no significant correlation between clinical variables (PANSS, YMRS and HDRS scores) and the measured M100 parameters in patients with schizophrenia and bipolar disorder.

**Table 3:** M100 latencies and strengths for each group.

	**Left hemisphere**		**Right hemisphere**	
	**latency (ms)**	**strength (nAm)**	**latency (ms)**	**strength (nAm)**
**Control**	113.18 (13.22)	21.96 (11.81)	111.05 (15.73)	20.07 (8.87)
**Schizophrenia**	113.02 (14.28)	15.10 (7.32)	110.20 (16.62)	15.24 (7.08)
**Bipolar disorder**	112.14 (16.40)	17.67 (11.19)	117.29 (19.62)	19.28 (11.24)

Standard deviations are in parentheses.

## Discussion

This is, to our knowledge, the first time the auditory M100 asymmetry has been compared directly in patients with schizophrenia, patients with bipolar disorder, and healthy control subjects. In the present study, the use of magnetic source imaging allowed localization of the auditory M100 to posterior STG or near primary auditory cortex in three groups. A M100 anterior–posterior positional asymmetry was present only in healthy controls, with schizophrenia and bipolar data indicating sources were more or less symmetrical between the hemispheres. These data suggest that schizophrenic and bipolar subjects may demonstrate partly similar disturbed functional asymmetry of temporal lobe, suggesting shared etiological and pathophysiological factors between the two disorders.

Some MEG-based studies found the auditory M100 components were localized to near Heschl’s gyrus and the planum temporale [22-24,28], supporting our findings of the M100 source localization to posterior STG. The STG, including the primary auditory cortex/ Heschl’s gryrus, planum temporale and planum polare cortices, plays pivotal roles in auditory, language, emotional processing and social cognition [29-31]. In the current study, the M100 source was located more anterior in the right STG region than in the left in normal control subjects, which is in agreement with previous studies [20,22,23]. A number of postmortem and structural MRI studies revealed right-left volumetric asymmetries of the STG, particularly the planum temporale, in normal adult and infant brains [32-34]. Chance and colleagues also reported minicolumn size asymmetries in the superior temporal lobe in normal subjects as a putative substrate of language processing [35]. This asymmetry may be largely attributable to greater white matter volumes, likely related to a greater number of fibers and/or increased myelination [35-37]. Additionally, Sowell and colleagues found temporal lobe asymmetry increased during adolescence and therefore was linked to hemispheric differences in white matter maturation [38].

In this study, absence of a right–left M100 anterior–posterior positional asymmetry for individuals with bipolar disorder was observed. This is the first report to our knowledge on the abnormal M100 asymmetry in bipolar disorder, and the results suggest the functional disorganization of the STG. Reite and colleagues found that bipolar patients had an abnormal asymmetry of steady state gamma band (SSR) in the auditory cortex, supporting the idea of anomalous functional laterality [26]. However, they failed to observe altered M100 source asymmetry. Their study used samples of euthymic bipolar patients (not meeting DSM-IV criteria for either mania or depression). Functional neuroimaging studies reported lack of normal pattern of hemispheric asymmetry in bipolar disorder [39]. Structural MRI studies also revealed reduced volumetric asymmetry in the STG [31,40]. Thus, our results together with these findings support the notion that structural/ functional asymmetrical alterations of the temporal lobe may play an important role in the pathophysiology of bipolar disorder.

Schizophrenia patients also showed a lack of normal M100 source asymmetry in the STG. These results are in agreement with some previous studies, which revealed reduction or even reversal of the auditory M100 asymmetry [22,23,25,41,42]. This altered asymmetry was mainly driven by group differences in the left hemisphere [23,43], whereas the right hemisphere was also reported to be deviant in schizophrenia [44]. In addition, Edgar and colleagues found the M100 hemispheric asymmetry index differed between schizophrenic patients and normal controls [45]. Their asymmetry index for the right and left M100 measurements was determined by subtracting the right from the left Y coordinates. However, the present study investigated M100 asymmetry index using the formula (R - L)/(R + L)×100 for the X, Y and Z coordinate did not differ among schizophrenia, bipolar disorder, and control group. Differences in the methodology (e.g., whole-head versus smaller channel arrays, planar versus axial gradiometers) and patient groups (e.g. gender differences, drug administration) may account for these reported discrepancies. In this study, we found the absence of interhemispheric asymmetry in schizophrenia (male/female 10/10) by using whole-head MEG, which was due to alterations of both right and left hemispheres. A number of MRI and postmortem studies found changed right–left temporal volumetric asymmetries in schizophrenia, usually consistent with a reduction in the left hemisphere and a relative increase in the right hemisphere [30,36,46]. Taken together, these findings suggest that individuals with schizophrenia present bilateral cortical disorganization. Chance and colleagues also indicated that disturbed STG asymmetry might be related to variation in the number of axons passing through the connecting regions of the corpus callosum [36], which support the notion that cortical misconnections underlie the symptoms of schizophrenia [47]. Additionally, Edgar and colleagues recently found temporal cortical thickness was associated with M100 auditory activity in schizophrenia [48], suggesting that functional abnormalities may be a consequence of elimination of the neuropil (dendritic arbors and associated synaptic infrastructure) between neuron bodies. 

The STG which is a key structure in language function may be particularly susceptible to developmental perturbations as the development of language is relatively recent and language areas may be only weakly canalized [49]. Schmidt and colleagues demonstrated an association between language functioning and M100 source asymmetry in children, suggesting a possible relationship between functional/ structural asymmetry of the STG and language ability [50]. Crow offered an account of schizophrenia is linked to disturbances in language function, especially involving the temporal lobe [51]. Two studies found that the anomalous M100 temporal lobes asymmetry in schizophrenia as well as dyslexia might be related to abnormal language development [43,45]. They hypothesized that reduced cerebral asymmetry of language fosters psychotic tendencies and thus developmental dyslexia with its altered hemispheric asymmetry might be a precursor for schizophrenia. Thus, anomalous auditory M100 asymmetry may represent an endophenotype shared among several neurodevelopmental conditions, which is not a diagnostically specific feature in schizophrenia or bipolar disorder. However, in this study, we did not explore the relationship between language function and auditory M100 asymmetry in patients with schizophrenia and bipolar disorder, further studies should examine these potential relationships.

To our knowledge, this is the first study to find partly similar M100 asymmetry abnormalities in the two disorders, suggesting anomalous functional asymmetry of the temporal lobe are shared by patients with schizophrenia and bipolar disorder. These findings indicated that schizophrenia and bipolar disorder are not completely dichotomous entities at least at the level of neuroanatomical phenotype, and they share common neurobiological abnormalities. These findings further supported the concept of a psychotic continuum, including schizophrenia, schizoaffective disorder, and both bipolar and unipolar affective psychoses, as has been suggested by Crow and others [52, 53]. Wilson and colleagues suggested aberrant cortical maturation processes contribute to the reduction in cerebral laterality commonly associated with psychosis [54]. Neuroatomical and neurochemical studies also demonstrated anomalous hemispheric asymmetries may reflect disturbances of the neurodevelopmental processes. Moreover, these disturbances may be influenced, to different extents, by genetic and/or environmental factors. These findings are consistent with the view that schizophrenia and bipolar disorder are a disorder of the genetic mechanisms that control the development of cerebral asymmetry. Additionally, recent studies proposed smoking may alter auditory microcircuits and thereby diminish left–right differences [55]. Thus, we recruited all participants who were non-smokers to reduce bias.

Some EEG-based studies have revealed reduced amplitude of the auditory N100 (or N1) component in schizophrenia, suggesting the N100 deficits might be a potential trait marker of schizophrenia [56,57], whereas negative findings have also been reported [57,58]. Alternatively, reports of N100 measures in bipolar disorder have been mixed [58,59]. However, in the present study, no abnormal M100 source strength and latency was observed in bilateral hemispheres in schizophrenia and bipolar disorder. In a review of studies examining N100 in schizophrenia, Rosburg proposed that a reduction of N100 amplitude in schizophrenia patients depend on ISI: N100 is more consistently reduced in studies using ISI >1 s than in studies using shorter ISI [57], supporting our results of normal M100 strength with relatively short ISI in patients. Furthermore, N100 (M100) amplitude is influenced by a number of other factors, such as stimulus intensity, arousal, selective attention, and drug administration [57,60,61].

We have shown that MEG provided good spatial/temporal resolutions for investigating schizophrenia and bipolar disorder, which is considerable strength of this study. However, some potential limitations of the present study should be taken in consideration. First, the relatively small sample size may have reduced the statistical power of our analyses. It is possible that a significant difference in M100 asymmetry index among groups would have been detected in a larger sample size. Second, the patients included had taken medicine prior to MEG and MRI scanning and it is difficult to ascertain the specific duration of drug treatment for each patient. Therefore, the effects of medication could be confounding factors in the analysis. We found no difference in the M100 latency and strength within three groups, which may be the variable most sensitive to medication effects. And finally, the inclusion of bipolar patients without a history of psychosis, schizoaffective disorder could have provided a more detailed picture of cerebral asymmetry abnormalities and a further understanding of the relationship between bipolar disorder and schizophrenia. 

## Conclusion

In conclusion, the present study reported patients with schizophrenia and bipolar disorder failed to show a right-sided auditory M100 anteriority found in healthy controls, suggesting dysfunction of temporal lobe in the two disorders. It was concluded that common disturbed M100 positional asymmetry might imply an overlap in STG pathophysiology in the two disorders and might be related to shared risk factors for the two disorders. These results challenge the current nosological dichotomy between schizophrenia and bipolar disorder, and are consistent with a reappraisal of these disorders as distinct diagnostic entities. Further analysis involving large numbers of scales of patients and using MEG combined with other neuroimaging techniques, such as voxel based morphometry (VBM), diffusion tensor imaging (DTI) or magnetic resonance spectroscopy (MRS), as well as with neuropsychological and genetic variables, would shed more light on our understanding of the etiology of schizophrenia and bipolar disorder.
